# FGF21 as a Hepatokine, Adipokine, and Myokine in Metabolism and Diseases

**DOI:** 10.3389/fendo.2014.00107

**Published:** 2014-07-07

**Authors:** Nobuyuki Itoh

**Affiliations:** ^1^Kyoto University Graduate School of Pharmaceutical Sciences, Kyoto, Japan

**Keywords:** adipokine, biomarker, disease, FGF21, hepatokine, metabolism, myokine, stress

## Abstract

Fibroblast growth factor (FGF) family members are mostly secreted as signaling proteins with diverse functions in development and metabolism. FGF21 is a unique FGF with metabolic, but not proliferative activities. FGF21 is mostly induced by different kinds of stress and acts though FGF receptor 1c with β-Klotho as a cofactor in an endocrine or, in parts, autocrine/paracrine manner. Hepatic FGF21 directly acts on white adipocytes to inhibit lipolysis and acts through the brain to increase systemic glucocorticoid levels and suppress physical activity in response to starvation. It also protects against dioxin toxicity. Adipocytic FGF21 induces the browning of white adipose tissue (WAT) and activates brown adipocytes in response to cold exposure. It also acts as an upstream effector of adiponectin in white adipocytes. Myocytic FGF21 protects against diet-induced obesity and insulin resistance, induces the browning of WAT, and protects against cardiac hypertrophy. In addition, *Fgf21* polymorphisms are possibly related with metabolic diseases and FGF21 are biomarker of metabolic diseases. These findings indicate that FGF21 plays roles as a hepatokine, adipokine, and myokine in metabolism, injury protection, and diseases.

## Introduction

Fibroblast growth factors (FGFs) are signaling proteins of ~150–300 amino acids with diverse biological functions mainly in development and metabolism. The human/mouse FGF family comprises FGF1–FGF23. However, as mouse FGF15 and human FGF19 are orthologs, they are usually referred to as FGF15/19 (1). FGFs can be classified into paracrine, intracrine, and endocrine FGFs based on their mechanisms of action. Paracrine FGFs mainly function as secreted local signaling molecules in development. Endocrine FGFs mainly function as hormone-like or, in parts, local signaling molecules in metabolism. In contrast, intracrine FGFs mainly function as intracellular molecules in neuronal processes (1).

Endocrine FGFs comprise FGF15/19, FGF21, and FGF23 (1). Although most of paracrine and endocrine FGFs have proliferative activities, FGF21 is a unique FGF with metabolic, but not proliferative activities. FGF21 exerts diverse pharmacological effects on metabolism. Review articles that focus on its pharmacological effects and therapeutic uses for metabolic diseases have already been published (2, 3). This succinct review focuses on the physiological and pathophysiological roles of FGF21 as a metabolic regulator.

## Mechanism of Action of FGF21

Paracrine and endocrine FGF signaling is mostly mediated by the activation of FGF receptors (FGFRs) including FGFRs 1b, 1c, 2b, 2c, 3b, 3c, and 4 with different ligand-binding specificities. FGFs activate four key intracellular signaling pathways: RAS/RAF/mitogen-activation protein kinase (MAPK), phosphatidylinositol 3-kinase (PI3K)/serine–threonine protein kinase AKT, signal transducer and activator of transcription (STAT), and phosphoinositide phospholipase C (PLC) γ (1). Endocrine FGFs activate FGFRs with α-Klotho or β-Klotho as a cofactor, which are specifically expressed in their targeted tissues (1). FGF21 activates FGFR1c with β-Klotho (1, 4).

## FGF21 as a Hepatokine

*Fgf21* is abundantly expressed in the liver. Hepatic *Fgf21* expression was possibly induced during fasting through the activation of peroxisome proliferator-activated receptor (PPAR) α by non-esterified fatty acids that are released from adipocytes and taken up by hepatocytes (5). Lipolysis was increased in the white adipocytes of fasted *Fgf21* knockout mice, indicating that FGF21 inhibit lipolysis during fasting (Table 1) (6). A low-carbohydrate, high-fat ketogenic diet (KD) significantly induced hepatic *Fgf21* expression. The impaired insulin sensitivity in WAT caused by KD feeding was improved in the knockout mice, indicating that FGF21 is a negative regulator of adipocyte insulin sensitivity in adaptation to a low-carbohydrate malnutritional state (7). FGF21 also increased systemic glucocorticoid levels and suppressed physical activity in adaptation to the starvation response. Mice lacking β-Klotho in the suprachiasmatic nucleus and dorsal vagal complex of the brain were refractory to these effects. Thus, hepatic FGF21 also exerts diverse actions through β-Klotho in the brain (Table 1) (8).

**Table 1:** **Functions of FGF21 as a hepatokine, adipokine, and myokine**.

FGF21	Targeted tissues	Functions	Action manners	Reference
Hepatokine	WAT	Lipid metabolism	Endocrine	(6)
	Brain	Glucocorticoid metabolism, physical activity, circadian behavior	Endocrine	(8)
	Not identified	Protection against dioxin toxicity	Endocrine	(9)
Adipokine	WAT	Thermogenesis	Autocrine/paracrine	(10)
		Adiponectin induction	Autocrine/paracrine	(11, 12)
	BAT	Thermogenesis	Autocrine/paracrine	(13, 14)
Myokine	WAT	Energy metabolism, thermogenesis	Endocrine	(15, 16)
	Skeletal muscle	Insulin resistance	Autocrine/paracrine	(17)
	Heart	Cardiac protection	Autocrine/paracrine	(18)

*WAT, white adipose tissue; BAT, brown adipose tissue*.

In addition to fasting and KD, hepatic *Fgf21* expression was significantly induced by different kinds of stress such as hepatic injury, chemical insult, and diseases. Hepatic FGF21 is a stress-induced metabolic regulator (19). The toxicity of dioxins is also well documented. Dioxins increased hepatic *Fgf21* expression and serum FGF21 levels. Its toxicity was enhanced in *Fgf21* knockout mice, indicating that FGF21 protect against this toxicity (Table 1) (9).

## FGF21 as an Adipokine

Uncoupling protein 1 (UCP1) releases chemical energy as heat in brown adipocytes. Beige adipocytes, brown adipocyte-like cells, are UCP1-positive adipocytes in white adipose tissue (WAT). Beige adipocytes markedly accumulated in subcutaneous WAT following cold exposure (20). Cold exposure induced *Fgf21* expression in WAT expressing *Fgfr1c* and β*-Klotho*. The accumulation of beige adipocytes was impaired in *Fgf21* knockout mice, indicating that FGF21 induces the accumulation of beige adipocytes in WAT in an autocrine/paracrine manner (Table 1) (10). Cold exposure also induced *Fgf21* expression in brown adipocytes expressing *Fgfr1c* and β*-Klotho*. FGF21 also activates brown adipocytes in an autocrine/paracrine manner (Table 1) (13, 14). In humans, cold exposure increased circulating FGF21 levels, which activated brown adipocytes and enhanced lipolysis and thermogenesis response through browning of WAT (21, 22). Thus, FGF21 activates the thermogenic machinery against hypothermia.

The systemic administration of FGF21 had no effect on metabolism in lipodystrophic mice with a WAT deficiency. However, the administration of FGF21 was effective in these mice following the transplantation of WAT (23). Thus, WAT is the predominant site that confers the metabolic activities of FGF21. Adiponectin, an adipokine produced in WAT, controls systemic glucose and lipid homeostasis in the liver and skeletal muscle in an endocrine manner. FGF21 has many functional similarities to adiponectin. Furthermore, FGF21 produced in WAT enhanced the expression of *adiponectin* in WAT as well as serum adiponectin levels. Several therapeutic benefits of FGF21 were impaired in *adiponectin* knockout mice. The effects of FGF21 on the attenuation of obesity-induced impairments in insulin signaling in the liver and skeletal muscle were also impaired in *adiponectin* knockout mice. Thus, adiponectin acts as a downstream effector of FGF21 in WAT and mediates the effects of FGF21 on energy metabolism and insulin sensitivity in the liver and skeletal muscle (Table 1) (11, 12). Insulin resistance develops in insulin-responsive tissues due to the aberrant accumulation of intracellular lipids including the sphingolipid ceramide. FGF21 diminished the accumulation of ceramides in obese animals. Changes in energy expenditure and the ceramide-lowering effects induced by FGF21 were impaired in *adiponectin* knockout mice. Thus, the FGF21–adiponectin–ceramide axis controls energy expenditure and insulin action (12).

## FGF21 as a Myokine

The PI3K/Akt1 pathway has been implicated in insulin signaling and cellular hypertrophy. Skeletal muscle fiber hypertrophy was observed in skeletal muscle-specific *Akt1* transgenic mice. The expression of *Fgf21* in the muscle and serum FGF21 levels was increased in the *Akt1* transgenic mice (24). Thus, skeletal muscle is also a source of FGF21, the expression of which is regulated by a PI3K/Akt1 signaling pathway-dependent mechanism.

FGF21 is also known to be a myokine that is induced by different kinds of stress (19). Cytoplasmic constituents are delivered to lysosomes for the degradation of aggregated proteins and recycling of organelles or nutrients by autophagy. The amino acids produced by autophagy are used for energy production or other purposes during nutrient deficiencies. Mitochondrial dysfunction in *autophagy-related 7* (*Atg7*) knockout mice increased *Fgf21* expression by activating transcription factor 4 (Atf4), a master regulator of the integrated stress response (15). An autophagy deficiency and subsequent mitochondrial dysfunction increased the production of FGF21 as a myokine to promote protection against diet-induced obesity and insulin resistance (Table 1). The induction of *Fgf21*, resistance to diet-induced obesity, and amelioration of insulin resistance were also observed in the livers of mice with autophagy deficiencies (15).

*Fgf21* was induced in the skeletal muscle of *Ucp1* skeletal muscle-specific transgenic mice and resulted in significantly elevated serum FGF21 levels. The integrated stress response was activated in the skeletal muscle of *Ucp1* transgenic mice without myopathy or a muscle autophagy deficiency. The browning of WAT with lipolysis and respiratory capacity was also increased in *Ucp1* transgenic mice (16). Improved substrate metabolism and increased longevity were also observed in *Ucp1* transgenic mice. Targeting mitochondrial function in cultured myoblasts by treatments with respiratory chain inhibitors resulted in the activation of an integrated stress response associated with the increased expression of *Fgf21*. In addition, white adipocytes cultured with the serum of *Ucp1* transgenic mice resulted in an increase in the expression of *Ucp1*. These findings indicate that the production of FGF21 is coupled to mitochondrial dysfunction and the activation of an integrated stress response in skeletal muscle. Thus, FGF21 as a myokine exhibits effects that lead to the increased browning of WAT in an endocrine manner (Table 1) (16). A subgroup of human immunodeficiency virus (HIV) patients is associated with insulin resistance. *Fgf21* expression in skeletal muscle, but not serum FGF21 levels was increased in HIV patients with lipodystrophy and this was correlated to insulin resistance, indicating that FGF21 is a myokine that may be correlated with insulin resistance in an autocrine/paracrine manner (Table 1) (17).

Exaggerated cardiac hypertrophy and dysfunction were observed in *Fgf21* knockout mice in response to the infusion of isoproterenol. Cardiomyocytes produced and secreted FGF21, indicating that FGF21 is a myokine that may protect against cardiac hypertrophy in an autocrine/paracrine manner (Table 1) (18).

## *FGF21* Single Nucleotide Polymorphisms as Risk Factors for Diseases

The intake of dietary macronutrients is associated with an increased risk of obesity and diabetes. A single nucleotide polymorphism (SNP) in the *Fgf21* exon was correlated with the percentage of total caloric intake from protein and carbohydrate, suggesting that *FGF21* is a potentially susceptible gene for obesity and diabetes (25). SNPs in the *Fgf21* 3′ non-coding region were also associated with metabolic syndrome, obesity, and diabetes (26) (Table 2). These findings indicate that *Fgf21* SNPs are possibly related with metabolic diseases.

**Table 2:** **FGF21 as a risk factor for and biomarker of diseases**.

FGF21	Diseases	References
**RISK FACTOR**		
SNP in the exon	Dietary macronutrient intake	(25)
SNPs in the 3′non-coding region	Metabolic syndrome, obesity, Type 2 diabetes	(26)
**BIOMARKER**		
Increased serum levels	Coronary heart disease	(27)
Increased serum levels	Carcinoid atherosclerosis	(28)
Increased serum levels	Obesity, type 2 diabetes	(29)
Increased serum levels	Mitochondrial disease	(30)
Increased serum levels	Cushing’s syndrome	(31)
Increased serum levels	Preeclampsia	(32)
Decreased serum levels	Anorexia nervosa	(33)

*SNP, single nucleotide polymorphism*.

## Serum FGF21 Levels as Biomarkers of Diseases

Coronary heart disease (CHD) with a narrowing of the small blood vessels in the heart could lead to heart attacks. Serum FGF21 levels were significantly higher in CHD patients, and were even higher in CHD patients with diabetes, and hypertension. However, high serum FGF21 levels were associated with adverse lipid profiles in CHD patients, indicating that this paradoxical increase in CHD patients may reflect a compensatory response or resistance to FGF21 (27). Carcinoid atherosclerosis typically results in carotid stenosis. Serum FGF21 levels were positively correlated with carotid intima-media thickness (IMT) in women (28). Serum FGF21 levels were also significantly higher in patients with both obesity and type 2 diabetes (29), in mitochondria disease patients with dysfunctional mitochondria (30), in Cushing’s syndrome patients with prolonged and inappropriately high levels of glucocorticoids (31), and in preeclampsia patients with serious cardiovascular complications in pregnancy (32). In contrast, serum FGF21 levels were decreased in anorexia nervosa patients with prominent reductions in body weight due to eating disorder (33) (Table 2). These findings indicate that serum FGF21 levels may be used as biomarkers of these diseases.

## Conclusion

FGF21 acts in an FGFR-dependent manner with β-Klotho as a cofactor. FGF21 with metabolic, but not proliferative activities has unique functions. FGF21, which is typically induced by different kinds of stress, plays various roles in energy metabolism and injury protection as a hepatokine, adipokine, and myokine in an endocrine or autocrine/paracrine manner. *Fgf21* single polymorphisms are possibly related with metabolic diseases and FGF21 and biomarker of metabolic diseases. These findings provide new insights into the physiological and pathophysiological roles of FGF21.

## Conflict of Interest Statement

The author declares that the research was conducted in the absence of any commercial or financial relationships that could be construed as a potential conflict of interest.
